# Monaco

**Published:** 1889-03

**Authors:** Joseph Simmes


### MONACO.

### THE GAMBLER'S PARADISE.

Monaco, on the Gulf of Genoa, is reckoned Italian, but is enclosed on the land side by French territory. Its independence dates from the tenth century, when its sovereignty was vested in the House of Grimaldi, with which it has since remained. It formerly measured 10 miles by 6, with a population of 7,000; but by various concessions to France the territory is now reduced to the town of Monaco and its environs, being an area of about eight square miles, with 12,548 inhabitants and an army of 126 men. There is a telegraph office and a postal establishment issuing its own stamps and employing letter carriers. The exports are olive oil, oranges, lemons, liquors, perfumes and pottery. The famous gambling establishment of Monte Carlo forms part of this little state, and pays a handsome sum yearly to the prince for the privilege of extracting from thirty to forty million francs out of the misguided votaries of this pleasure. Besides the eight large tables for roulette, Trente et Quarante and other games, which form the staple, there is a bank, a reading room and a theatre, with free concerts of excellent music by fifty or sixty performers every evening. In short, this gambling company has spared nothing that human ingenuity could devise, in order to attract visitors and lure them to their ruin. Here, as is well known, men and women play with infatuated earnestness, hoping for gain, and not seldom, when a man has lost his all, he repairs to a neighboring hotel and puts an end to his life. There are usually from two hundred to three hundred players, and the games are kept up from eleven in the morning till eleven at night. We counted nine bald heads among the younger ones around one table, and thirteen gray haired men at another. One would have thought that gambling at this rate would have been left to the young and sanguine ; but there are old fools as well as young ones, ready to become the dupes of designing men. If there is a literal hell in Europe, it is here, within the precincts of this little, corrupt principality. The shareholders have been informed that the Casino never had so profitable a season as the last has been, and the report adds this item—seventy-six suicides. The latest we have heard of is probably not included, as it occurred on December 27 last and was reported to a Scotch newspaper by a person on the spot. A young, handsome and once respectable female, who went to Monte Carlo some years ago, ran through the too common course. The passion for gambling developed in her rapidly. She frequented the tables daily ; she lost and won ; played more and more rashly, and as her gold diminished, supplemented her resources by a life of shame. On the Tuesday after Christmas she carried her jewels, not for display on her person, but in her pocket, to obtain money from the "stranglers," as those hangers-on are called, who give advances on such property. At once she took her place at the roulette table, and at nightfall was a beggar. She entered her hotel about midnight, went straight to her bedroom, and was found next day hanging from the pole of the window curtain. The authorities of the Casino were sent for immediately, a fact which shows that they accept the responsibility of such occurrences. They came at once, cut down the body, settled with the hotel keeper, cleaned up the room and buried the corpse in their beautiful garden—an "Aceldama," where lie the remains of many suicides from various lands. In Monaco, there are no coroner's inquests, or other like investigations ; no communication with the relatives of the deceased. The bodies are consigned to unlettered graves ; and who they were or what they were is hushed up by the authorities. And why? The whole principality, from the prince to the scavenger, lives by the Casino, with all its dreadful tragedies.—*Extract from letter of Dr. Joseph Simmes.*

